# Experiences and Perceptions About Death Reporting and Notification Among Rural Communities on the Islands of Lake Victoria, Uganda: Qualitative Study

**DOI:** 10.2196/77135

**Published:** 2025-08-13

**Authors:** Steven Ndugwa Kabwama, Douglas Bulafu, Rawlance Ndejjo, Rose Nampeera, Christine Kihembo, Chukwuma David Umeokonkwo, Simon Antara, Sheba Nakacubo Gitta, Caroline Kyozira, Allan Muruta, Jean-Edgard Nguessan, Rhoda K Wanyenze

**Affiliations:** 1 Department of Global Public Health, Karolinska Institutet, Tomtebodavägen 18A, Solna, Stockholm, 17177, Sweden, 46 7 075 780 93, 46 8 524 800 00; 2 Department of Community Health and Behavioral Sciences, School of Public Health, Makerere University, Kampala, Uganda; 3 Cluster of Research Excellence for Preparedness and Response to Pandemics and Shocks, Kampala, Uganda; 4 Department of Disease Control and Environmental Health, School of Public Health, Makerere University, Kamplala, Uganda; 5 Department of Preventive Medicine, College of Medicine, Korea University, Seoul, Republic of Korea; 6 Department of Programs, African Field Epidemiology Network, Kampala, Uganda; 7 Department of Epidemiology and Biostatistics, School of Public Health, Makerere University, Kampala, Uganda; 8 Department of Integrated Epidemiology, Surveillance and Public Health Emergencies, Ministry of Health, Kampala, Uganda; 9 Department of Noncommunicable Diseases, CDC Foundation, Atlanta, United States

**Keywords:** mortality, surveillance, death notification, death reporting, Uganda

## Abstract

**Background:**

Mortality data are critical for planning and prioritization of public health interventions and are generated through civil registration and vital statistics systems like mortality surveillance systems. However, frameworks for strengthening mortality surveillance systems do not acknowledge the cultural relativism surrounding death and how it influences strategies to improve mortality surveillance systems.

**Objective:**

This paper aims to describe the experiences and perceptions about death reporting and notification among rural dwellers on the islands of Lake Victoria in Central Uganda.

**Methods:**

The study was conducted in Buvuma and Kalangala Districts on Lake Victoria using a phenomenological qualitative research design. We conducted 12 in-depth interviews with community members who were purposively identified by village leaders and had experienced the death of a next of kin and reported and notified, and 8 in-depth interviews with those who had experienced the loss of a next of kin but did not notify and report the death. Key informant interviews were also conducted with 2 police officers and 2 cultural leaders. A total of 4 focus group discussions were conducted among village leaders. Interviews were abductively analyzed to generate grand narratives.

**Results:**

The findings revealed 6 grand narratives of the perceptions and experiences of the process of death reporting and notification among the rural dwellers. These include (1) death reporting and notification are preceded by a tragic event that affects how, when, and if it is conducted; (2) a long and cumbersome process; (3) a process that involves multiple stakeholders with official and unofficial roles and responsibilities; (4) a process with little perceived individual or societal value; (5) a process with several mandatory but unofficial costs; and (6) a process preceded by events with deep cultural undertones.

**Conclusions:**

Death reporting and notification are perceived to be tedious and cumbersome, which discourages community members from conducting them. There is a need to evaluate the process to remove any perceived or actual barriers through strategies such as decentralization of the process to lower levels of political administration. Death reporting and notification are also part of a broader social context that includes cultural beliefs, norms, and traditions. Efforts to strengthen mortality surveillance systems would profit from acknowledging the broader sociocultural issues around death and grieving and the role that cultural and religious institutions can contribute to addressing misconceptions and articulating the benefit of the process to society.

## Introduction

Mortality data are critical for planning, monitoring, and evaluating the implementation and impacts of national health policies and programs [1]. The data can be analyzed to identify changes in disease burden due to specific public health policies or interventions that can inform reprioritization and reallocation of resources. Mortality data are generated through national civil registration and vital statistics (CRVS) systems that provide information on death by sex, age, and cause of death. Ecological analyses have shown that good CRVS systems are associated with better population level health outcomes such as lower risk of child mortality, lower maternal mortality ratio, and better healthy life expectancy [2]. However, many low- and middle-income countries, particularly in Sub-Saharan Africa, lack comprehensive CRVS systems [3]. According to the 2022 United Nations Populations and Vital Statistics Report, many low- and middle-income countries, including Uganda, did not report any vital statistics such as live births and deaths in the 15 years of the reporting period between 2006 and 2020 [4]. Although some countries have made progress in improving maternal and perinatal death surveillance [5,6], challenges remain regarding the establishment of mortality surveillance systems that comprehensively record all deaths that occur outside of health facility settings. The 2016 Uganda Demographic Health Survey noted that only 24.2% of all deaths that occur in the country are reported, notified, and registered [7].

In 2023, the Africa Centres for Disease Control and Prevention (Africa CDC) published a continental framework for strengthening mortality surveillance systems in countries in Africa [8]. The framework contextualizes the mortality surveillance process by emphasizing the systematic collection of death-related data through leveraging community structures to implement an active case search approach and linking the information to policy and decision makers for public health action. It specifies the core functions of mortality surveillance systems at various levels of the health system, such as the detection of events and notification to national civil registration systems at both the health facility and community level. However, aside from the policy and infrastructural investments required to improve mortality surveillance systems, the framework does not acknowledge the cultural relativism [9], surrounding death or the wide societal and cultural variations in the interpretation, articulation, and meaning of death as a concept, and the bereavement process [10], and how these relate with, and influence strategies to improve mortality surveillance systems on the continent. From an anthropological standpoint, mortality is associated with grief, mourning, and bereavement, which are complex social phenomena shaped by deeply entrenched cultural norms, attitudes, and beliefs about death and the dead [11]. These norms, attitudes, and beliefs can influence how the process of death reporting and notification is perceived, interpreted, experienced, and conducted. Island communities often have unique cultural identities [12] and logistical challenges to implementing public health interventions [13] like surveillance. We conducted a study to describe what death reporting and notification mean for rural dwellers on the islands of Lake Victoria in Central Uganda to inform strategies for aligning and contextualizing interventions to improve mortality surveillance systems in Africa.

## Methods

### Study Area

**Figure 1.**
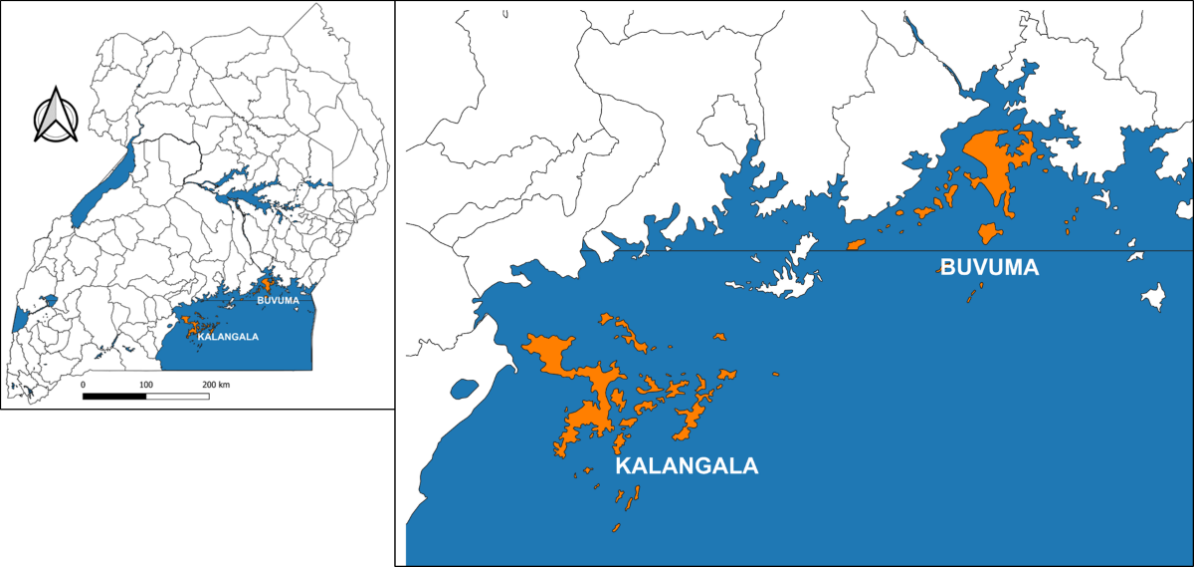
Map showing Buvuma and Kalangala Districts on Lake Victoria, Central Uganda.

The study was conducted in Buvuma and Kalangala Districts on Lake Victoria (see Figure 1). Buvuma District comprises 9 subcounties on 52 islands with a population of about 120,000 people, while Kalangala District consists of 7 subcounties on 84 islands and a population of about 70,000 people [14]. Buvuma and Kalangala Districts are among the least populated districts in Uganda and have been described as hard-to-reach [13] because of their geographical location on Lake Victoria, which presents unique challenges in terms of service delivery, including access to health services.

The islands are part of the Buganda Kingdom in Central Uganda. The kingdom is a constitutional monarchy composed of 52 clans and headed by a king (Kabaka), who is supported by a prime minister (Katikkiro), a legislative assembly, and leaders at the county, subcounty, and parish levels. The subjects of the kingdom are called Baganda and have cultural norms and traditions related to language, family, identity and genealogy, fashion and food, spirituality and beliefs, and ceremonies such as marriage, birth, and death.

### Study Design

The study used a phenomenological qualitative research design [15,16], which aimed to distill the perceptions and lived experiences of individuals in a rural community regarding the process of death reporting and notification. The choice of this study design was based on social constructivism as a knowledge claim [15], acknowledging the cultural relativism [9], and the various contexts where people live and work that determine and shape the experiences of the process of death reporting and notification.

### Study Population

The study population was the inhabitants of Buvuma and Kalangala Districts. The study participants were community members who had experienced the death of a next of kin and were purposively selected by village leaders. The village leaders are part of the local administrative structure that oversees public health, education, and social welfare [17], and participate in all burial ceremonies in their respective villages.

### Sampling and Sample Size

In-depth interviews were conducted with 20 community members who had experienced the death of a next of kin in the 12 months that preceded the date of the interview. These included 12 community members who had experienced the death of a next of kin and reported and notified the death as well as 8 community members who had lost a next of kin but did not notify and report the death. Community members who were sick, ages 18 years and younger, or could not provide consent for the interview were excluded from the study. Table 1 shows the characteristics of participants included in the in-depth interviews.

To enhance the trustworthiness of the study [18,19], focus group discussions (FGDs), and key informant interviews (KIIs) were also conducted. A total of 4 FGDs were conducted among local administrative leaders with each group comprising 8‐10 participants. Key informant interviews were also held with 2 police officers and 2 cultural leaders at the subcounty level. The KIIs were equally distributed across the 2 districts, with at least one in each of the selected subcounties. The selection of the police officers and cultural leaders at the subcounty level was purposive as stakeholders involved in funeral ceremonies [20].

**Table 1.** Characteristics of the in-depth interview participants.

Characteristic	Reported and notified (n=12), n (%)	Did not report (n=8), n (%)
Sex		
Male	1 (8)	3 (38)
Female	11 (92)	5 (62)
Age categories (years)		
20‐29	0 (0)	1 (13)
30‐39	3 (25)	2 (25)
40‐49	4 (33)	2 (25)
50‐59	4 (33)	2 (25)
60+	1 (8)	1 (13)
Education level		
No formal education	4 (33)	3 (38)
Primary education	6 (50)	4 (50)
Secondary education	2 (17)	1 (12)
Tertiary education	0 (0)	0 (0)
Employment		
Peasant or farmer	12 (100)	8 (100)

### Death Reporting and Notification in Uganda

The Uganda National Identification and Registration Authority (NIRA) published a death registration handbook [20], which outlines the steps of reporting, notifying, and registering a death. Briefly, when a death occurs in the community, the deceased’s next of kin obtains a recommendation letter from the administrative leadership at the village level. The next of kin then takes this letter to the administrative leadership at the subcounty level for validation and issuance of a notification record. Thereafter, they take the notification record to the NIRA district office where the death is registered and subsequently a death certificate is issued.

If the death occurs in a health facility, the health worker fills out the death notification record and then a medical officer fills the medical certification of cause of death. A records officer enters this information into the national health information system which is linked to the national information and registration authority database. The next of kin then goes to the NIRA district office to obtain a death certificate.

For both processes, the death certificate issued by NIRA is free if it is requested within a year of the death of the next of kin. Beyond this, the certificate is issued after payment of 5000 UGX, equivalent to US $1.40.

### Data Collection

The in-depth interview guides were developed following the constructs of the socioecological model [21]. The model acknowledges that actions such as engaging in notifying and reporting a death are influenced by factors that occur at the individual level (eg, knowledge about the process), at the interpersonal level (eg, a family member who knows the process), at the community level (eg, how cultures and traditions align with the requirement to notify and report death), and at the policy level (eg, the legal and policy environment for reporting and notification). In addition, reference was made to the Uganda NIRA Form 12 for community death notification and the HMIS Form 100 for health facility death notification to obtain information regarding the various steps and stakeholders involved in the reporting and notification process.

The guides for the key informant interviews, FGDs, and in-depth interviews were pretested through continuous role plays among the research assistants. This strategy has been used in previous studies and served to assess the clarity of the translations, skip patterns, and comprehensiveness of the guide to address all research objectives. The in-depth interviews, key informant interviews, and FGDs were conducted within the community at a place convenient for all interviewees. The key informant interviews and in-depth interviews lasted an average of about 40 minutes, while the FGDs lasted an average of about 60 minutes. The KIIs and FGDs were conducted by 6 female research assistants and 5 male research assistants who had at least Bachelor’s Degree training and were fluent in Luganda—the locally spoken language in the region. In addition, interviewers received additional training on the principles of research ethics as well as qualitative data collection techniques such as probing and taking important field notes to supplement the analysis. SNK, DB, and Ndejjo R held regular meetings with the research assistants to discuss and address any challenges regarding participant recruitment and the emerging themes as well as the rate at which new information was being generated. The data collection process was halted after SNK, DB, and Ndejjo R discussed with the research assistants and concluded that saturation was reached.

### Data Management and Analysis

All interviews and FGDs were audio recorded and transcribed verbatim. All transcripts were then entered into ATLAS.ti (version 8.0; Lumivero) and analyzed thematically. We used an abductive approach to data analysis, which involved both deductive and inductive processes. The first and second author gave an overview of the analytical approach to the team that conducted the analysis. A total of 8 people who were involved in the data collection supported the generation of codes and themes from the data. This allowed for researcher triangulation where the codes and themes were compared across the analysts to generate a narrative. Subsequent meetings were held to discuss the application of the framework and any improvements to improve the synthesis and generation of themes. Deductively, we generated themes following the Socioecological Model [21] as a guide, but with the flexibility to include themes and codes beyond the framework. We then merged these themes into several grand narratives [22] to construct the meaning of death reporting and notification based on the experiences and perceptions of the study participants. Grand narratives are broad descriptions of a phenomenon. In our case, we generated broad descriptions of what emerged as experiences and perceptions as told by our participants.

To enhance the completeness with which the methodological approach has been described, we have filled the Consolidated Criteria for Reporting Qualitative Studies checklist (see Checklist 1).

### Ethical Considerations

The study received ethical approval from Makerere University School of Public Health Research and Ethics Committee (Approval SPH-2024‐553). The study was also registered by the Uganda National Council for Science and Technology (HS3966ES). Permission to conduct the study in the selected districts was also obtained from Buvuma and Kalangala District Local Government authorities. Previous written informed consent was obtained from all study participants and participation was voluntary after establishing rapport and explaining the rationale for conducting the study. No individually identifiable information was collected, and each participant in the study was assigned a number to protect their identity. Each participant in the interviews was reimbursed with US $14, while those in the FGDs were reimbursed with US $6, to compensate their time for participating in the study. Only research assistants with a certificate in human subjects’ protection or good clinical practice participated in the study.

## Results

### Participants and Analysis

The study involved 56 participants. These included 12 participants in the in-depth interviews, 4 participants in the key informant interviews, and 40 participants in the FGDs.

The analysis of results from the interviews and FGDs revealed 6 grand narratives of the perceptions and experiences of the process of death reporting and notification among the rural dwellers in our study.

### Death Reporting and Notification Is Antedated by a Tragic Event That Affects How, When, and if it is Conducted

In-depth interview participants expressed that their experiences associated with death, loss, and the grief by individuals and families determine whether they conduct the process of death reporting and notification. Relatedly, the manner in which the death occurs also determines how it is reported and notified. For example, participants expressed feelings of shock and inertia when the death occurred suddenly. Initiating the process of reporting and notification was impeded in a context of sudden shock, grief, and mourning. Others noted that in circumstances where the death event is sudden and the cause is unclear, participants felt that initiating the process of death reporting and notification might imply guilt and culpability on the part of the notifier. In fact, there was a general lack of knowledge of who, how, and when to initiate the process of death reporting and notification.

*We experienced the death of my son who was involved in an accident with his friends while riding a motorcycle… I didn’t notify my son’s death because I couldn’t think straight at the scene of the accident. I simply informed the local leaders of the area. I also didn’t know what was required to register a death*

[In-depth interview, IDI 02, Did not report and notify]

Similar sentiments were expressed by participants who had experienced the death of a next of kin from a health facility:

[*my sister] needed to return to the health facility to acquire a specific form required by NIRA for the certificate. However, due to this grief, the process has been slow. As a result, we have not yet finalized obtaining the death certificate…*

[IDI 03, Reported but did not notify]

### Experience: A Long and Cumbersome Process

Participants noted that they found the process of death reporting and notification to be very long and cumbersome. In addition to the logistical challenges of movement in the context of the area being an island, participants noted that there were many offices one needed to go to and demanded too much in terms of time and financial resources. For example, the process involves organizing and attending family meetings, getting letters from the administrative leadership at village level, the notification form from the health facility, which are then taken to the administrative offices at the subcounty level.

*It’s a long process, full of bureaucratic systems, at all levels, the hospital, the community, police letters, clan meetings, and NIRA also takes time. So, the process is too long... the officers are often too busy to attend to death registrations. They require lots of confirmations from family members which would be difficult to collect from all the family members at a single time*.

[KII cultural leader 01]

Relatedly, participants noted that the death reporting and notification process required several documents and letters whose content and format were not clear. For example, participants noted that the process starts with a family meeting whose minutes are required for the administrative leadership to provide a recommendation letter. However, little guidance is provided for who should be in the family meeting and what the minutes should describe. Furthermore, the village leader (lowest administrative level leadership) provides a recommendation letter but little guidance is provided in terms of the format or the content of the letter.

*When I lost my brother last year, I had not heard anything about the death notification process. I didn’t even know where to start. I believe it’s important to complete the notification process because I’ve lost six family members, yet no one outside our immediate circle is aware of it.*

[IDI 04, Did not report and notify]

### A Process Involving Multiple Stakeholders With Official and Unofficial Roles and Responsibilities

Participants in the interviews described the death reporting and notification process as involving several stakeholders such as the family, relatives, friends, and clan members of the deceased, clan leaders and elders, administrative leadership at village and subcounty level, community health workers, and the police. The family and clan leaders have the responsibility of ensuring that cultural norms and values are followed in the funeral processes, the administrative leadership at the village level provides a letter of support that accompanies the death notification form, while community health workers provide technical support to community members going through this process. However, the role of the police and the administrative leadership at the subcounty level was unclear as noted by a respondent in the quote below:

*The problem with police is that it deals with those who are able to give them money in exchange for a service! If you don’t have money, they will ignore you!! So if this police we have were the conventional type where every person is listened to equally and fairly, collaborating with them on matters of death registration in the community would be a very good thing. Otherwise, as of now, we fear to interact with the police.*

[KII cultural leader 02]

### A Process With Little Perceived Individual or Societal Value

Generally, participants expressed little appreciation of the value of notifying and reporting a death. The general sentiment was that this was a process that was required if the deceased left an estate to be administered by a surviving next of kin. In circumstances of the death of a minor or where the deceased did not leave any estate, there was no motivation from the next of kin to report and notify the death. Participants felt that in addition to being cumbersome, there was no real direct benefit at both individual and societal levels.

*I saw no value to register that death; my person died… there was nothing to gain whatsoever. Now, my person was dead and the sub-county is far away! Why should I incur costs on transport to register this death yet I have children to provide for sugar and school fees?*.

[IDI 03, Did not report and notify]

Other participants perceived that death reporting and notification were only important in circumstances where the deceased had property because of the need to have an administrator who will take care of that property.

An FGD participant mentioned that:

*People who register are the people who have money and hope to get other property but for those without property, after burial; it is over and even forget about the name. …not all who lose people come to register their people but those who have need or when the deceased had some property. In most cases, if they have not called us to join their meetings, that person comes with a family letter confirming that he/she was selected to work on the deceased’s property*.

[FGD among local leaders]

### A Process With Several Mandatory but Unofficial Costs

Interview participants noted that the process of death reporting and notification involved several mandatory steps that required unofficial costs. For example, the process begins with a letter of support from the administrative leadership at village level. This letter is seldom given free of charge. The administrative leaders argue that the payments made are to support the procurement of stationery for issuing the letters. Similarly, the involvement of the police, particularly for deaths that involve accidents like drowning, will attract a cost on the part of the person trying to notify and report that death.

*We lost our dear child who had been suffering from an illness. We needed a support letter from the chairman confirming that the child had died at home… The chairman provided the letter without any issues as we had already informed him about the death. We also obtained a support letter from the police detailing the cause of death, the date, and the place of residence. At the chairman’s place, we paid US $1.4 for the letter. However, at the police station, they initially asked for US $5.5, which I couldn’t afford. I told them I could only manage US $2.8, and they accepted that amount.*

[IDI 06, Reported but did not notify]

Worse still, the communities in the 2 districts were generally poor, and any official or unofficial costs associated with death reporting and notification will only discourage anyone from following through with it. Death reporting and notification involves meeting different stakeholders including the village local council chairperson, the health facility, and NIRA officials, among others. Some of the relatives of the deceased were too poor to afford these costs associated with death reporting and notification.

### Death Reporting and Notification Are Preceded by Events With Deep Cultural Undertones

Participants noted that death and burial of the deceased were cultural events with several rites and rituals involving members of the family, community, and clan as well as cultural leaders. They noted the importance of adhering to prescribed cultural norms and practices when grieving and mourning the death of a loved one. Others were concerned that death reporting and notification was contrary to traditionally accepted cultural practices and might disturb the peace of the deceased, which discouraged some next of kin from reporting and notifying deaths.

*As per the culture here, they will say; Leave the person to rest, leave the person to rest. Why do you disturb him?*.

[KII Police Officer]

These sentiments were expressed by clan leaders and representatives of cultural leaders.

*“Because people don’t know as the Baganda say “Omuffu tebamubala” meaning “the dead cannot be counted”. So, they take registration of deaths as “counting the dead” people will be like why should you register a dead person? ...why do you make a death notification…*

[KII cultural leader 01]

## Discussion

### Principal Findings and Comparison With Previous Works

This study aimed to describe the meaning of death reporting and notification among rural dwellers on the islands of Lake Victoria in Central Uganda to inform strategies for aligning and contextualizing interventions to improve mortality surveillance systems in Africa. Our results revealed that death reporting and notification is preceded by a tragic event that affects how, when, and if it is conducted, a long and cumbersome process, which involves multiple stakeholders with official and unofficial roles and responsibilities, a process with little perceived value, involving several costs, and yet it is preceded by events with deep cultural undertones. The fact that participants felt that the death reporting and notification was difficult to initiate and conduct emerged as an experience among some groups and a perception among others. The challenge of complex systems and processes for reporting and notifying deaths has been reported in other countries like Mozambique [23] and Gambia [24] and has been exacerbated by poor logistical infrastructure coupled with little investment in technology, equipment, and human resources. The fact that this theme emerged from informants who had attempted to conduct the process, as well as those who only perceived it to be difficult, calls for a systematic approach to identify gaps and recommend adjustments for improvement. First, we propose a review of the entire process to clarify the contribution and role of each step, identify bottlenecks and opportunities for improved efficiency, and the subsequent removal of steps with unclear roles. In addition to addressing the practical or logistical difficulties of conducting the process, there is a need to improve how the process is appreciated and interpreted by the public. There are several theories in behavioral science and psychology that incorporate constructs of perception and motivation as indispensable precursors to action. For example, self-efficacy theory [25] posits that the belief that a task is difficult disincentivizes attempts to act. Perceptions and beliefs about the difficulty of a task are also critical in the psychological theory of cognitive dissonance [26], where the human mind engages in a struggle to align perceptions and beliefs with behavior. Psychological discomfort (dissonance) occurs where attempts are made to engage in an action that the mind perceives as difficult, resulting in inaction as the behavior to alleviate this discomfort. Relatedly, knowledge has been described as an indispensable precursor to perceptions [27] and beliefs. Interventions to improve awareness about and appreciation of the process of death reporting and notification in these communities should entail a clear articulation of its value to individuals, societies, and the health system. Such strategies could involve provision of specific examples of how mortality data are used to inform public health actions like identification of disease hot spots, planning, monitoring, and evaluation of interventions, policies, and health programs [28]. People are more likely to engage in tasks that they perceive to have high use value [29] for themselves, the community, or society.

Our study found that the process of death reporting and notification was described as one that involved multiple officials and stakeholders that were both official and unofficial. In addition, the process attracted several charges, most of which were unofficial. These 2 results corroborate our earlier findings that the process was perceived to be cumbersome. Aside from a review of the roles and relevance of the various steps, we propose further decentralization of the death reporting and notification process to the lowest levels of the political administrative structure. Here, a next of kin would report and notify a death at the village administrative officer or the parish administrative officer instead of the subcounty administrative officer. Although complex to implement [30], decentralization would alleviate the logistical challenges of movement that are particular to the communities in this context by reducing the administrative approvals one had to have before completing the process of death reporting and notification.

Participants also noted that death reporting and notification are preceded by a series of other norms and rituals with profound cultural significance. Efforts to improve mortality surveillance systems should avoid compartmentalizing the death reporting and notification process by ignoring the cultural significance of the events that precede it. Many cultures in Africa have norms and rituals that relate to recognizing the change of the body from physical to spirit form and ensuring that a good relationship remains between the spirit of the dead and the living [31]. If these processes are not properly conducted and followed, there are beliefs that the dead can have powers over and negatively impact the lives of the living [31-33]. In fact, the reservations about conducting the death reporting and notification process expressed by the participants in our study stemmed from concerns about offending the dead. Participants expressed sentiments about the cultural inappropriateness of talking about the dead or counting them, which discouraged some next of kin from conducting the process. Interventions and strategies to improve mortality surveillance systems on the continent should recognize the role of traditions and cultures and how these influence the understanding and appreciation of death reporting and notification. This can be operationalized through partnering with traditional and cultural institutions and leaders to promote death reporting and notification and its relevance to demystify the process and remove cultural barriers as one of the strategies for strengthening mortality surveillance. Acknowledging cultural norms and values to advance public health goals has been successfully used to improve access to maternal health services in Indonesia [34], and scale up male circumcision in Lesotho and Swaziland [35]. Policies to improve mortality surveillance should be sensitive to cultural nuance by recognizing the culture, people, food, language, and other contextual factors within which the policy is developed [36].

The findings from this work should be interpreted while considering some limitations. First, we included participants who described their perceptions and experiences with death reporting and notification within the year that preceded the interview. This could introduce some information bias regarding the comprehensiveness with which events could be recalled. However, we recruited research assistants with the relevant experience in conducting qualitative interviews and used techniques such as probing to enhance the completeness with which events were recalled. Also, the results presented in this paper are synthesized from multiple sources which improves the credibility of the research. In addition, the authors from the Ministry of Health could have introduced reflexivity bias when describing the challenges with the process of reporting and notifying deaths. However, these authors were not involved in the data collection and analysis process. The other limitation from this study could be the overrepresentation of females among the in-depth interview participants. This could imply that the results regarding the experience of death reporting and notification lacked generalizability to both males and females. However, the rationale for selecting participants was solely on the experience of having lost a next of kin within the year that preceded the interview and the gender imbalance in the participants was not intentional from the researcher’s perspective. Additional research would be required to assess whether there is nuance that was missed due to the gender imbalance in the in-depth interview participants.

### Conclusions

Death reporting and notification is an initial step in efforts to collate health-related data that is used for planning and evaluating health programs. Our study in this rural setting on the islands of Lake Victoria has established that the process is perceived to be tedious and cumbersome, which discourages community members from reporting and notifying deaths. There is a need to reevaluate the process to remove any perceived or actual barriers through strategies such as decentralization of the entire process to lower administrative levels. Death reporting and notification are also seen as being part of a broader context that includes cultural beliefs, norms, and traditions. Efforts to strengthen reporting and notification should acknowledge that death is a sociocultural issue and actively involve cultural and traditional institutions. This will be particularly relevant in efforts to dispel myths and misconceptions about death reporting and notification as well as articulating the benefit of the process to society.

## Supplementary material

10.2196/77135 Multimedia Appendix 1 Analyzed data.

10.2196/77135 Checklist 1 COREQ (Consolidated Criteria for Reporting Qualitative Studies) checklist.

## Abbreviations

Africa CDC: Africa Centers for Disease Control and Prevention
CRVS: civil registration and vital statistics
FGD: focus group discussion
KII: key informant interview
NIRA: Uganda National Identification and Registration Authority
