# Supplementary material for: Experiences and Perceptions About Death Reporting and Notification Among Rural Communities on the Islands of Lake Victoria, Uganda: Qualitative Study
Source: JMIR Public Health Surveill. 2025 Aug 13;11:e77135. doi: 10.2196/77135 (PMC12347208; doi:10.2196/77135)
Supplement: Multimedia Appendix 1 [file publichealth-v11-e77135-s001.pdf]

S/N	Themes	Subthemes	Short description/definition
		Property ownership	To show proof of ownership of property in order to be compensated.
		primary responsibility to care for my children	As the deceased's wife, I am securing a letter of administration to protect my children's inheritance from relatives' land grabs.
		Manage the deceaseds property	Death certificates are needed in order to get letters of administration to be able to manage the deceaseds estate.
		To seek justice	Some people may register deaths of their loved ones because they want to acquire justice for the death
		Determination to have children inherit prpoperties from their father	
		To receive assistance	
		Ownership of Property	Certification is only done for people who had properties and they have to place his properties in the right full owners/ administratrors
		The "will" of the deceased	The deceased will that is written helps to find position/ property administrators to help and support the process of death c ertification "
	Finance	selling property	Wanting to sell the deceased's property
	culture	Carry remains	Some come looking for their dead relatives to carry the remains back to their ancestral homes
	Government	Government policy	It is a policy to register all deaths that are transported and not burried in the area
		Proper planning	Government needs to know the population to plan well
		Compesation	Land was taken by government but awaits compersation
	Family	Family feuds	Misunderstandings among family members on ownership of proprty
		Family responsibility	its upon the family or relatives to process the death certificate of their loved ones to prove ownership of property
		Property owneship	Changing ownership of property
	Compensation		For the family members of the deceased to get compensated
	property management		manage property of the deceased

	Compensation/pension		To get benefits of the deceased
	Others	Tranportation of the body	The need to transport the body across the water is a great motivator for people to obtain the death certificate.
	Financing	Compensation	Most people are motivtaed death certificates of the deceased inorder get compensation fee from the responsible entities.
		Desire for letters of administration	It was noted that people would go ahead to process death certificates because they wanted to get letters of administration for the deceased's property
		Process money for the deceased	Family members were motivated because they wanted to be able to access money that the deceased was entitled to
		Waiver of bank loans	Banks could be in position to forgive an outstanding debt if there is evidence of the person's death
	Interpersonal	Property claims	A death certificate is needed in order to take over the deceased’s property
		Death registration upon request	LCs issue a notification letter upon request from the deceased's family, listing property and heirs.
	Cause & prevention of deaths		To help government know the causes of death in the community, the number of dead people and come with preventive measures to cub death
	Identity	Identity of the deceased	To keep the right identity of the deceased since some people come to the islands alone without any relatives
	property	Manage deceaseds money and property	People register deaths to be able to acquire money and property left behind by the deceased
	Others	Ascertainment of cause of death	Participants noted that what would motivate them to go ahead and register a death was the need to ascertain the root cause of death
	Health information	Description of mortality trend	Death registration would allow to have accurate figures of deaths that are happening in the community
	People/individual	Education level of the deceased's loved one	The educated do not need a reason to register death. They understand that it is a legal requirement and know the value of a death certificate in future investments etc
		Nature of work of the deceased	Government officials save pension and NSSF which can only be accessed by the family/loved ones if they have a death certificate

	Governance and leadership / policy	Legal safety	Registering death keeps one on the good isde of the law. The loved one is protected because they were not responsible for the deceased's death. The certificate entails the casue of death
		Legal requirement	For government officials, the deceased should be certified to regulate inflow and outflow of workers from the government system. Also important when inheriting admisnistrative power in businesses
		Limit ghost payments	Government to stop paying salaries of people who died
	Community / Culture	Transportation of the dead	The dead are always burried in their ancestral homes which are far from the place of death such as hospital or urban home. To transport the dead without hindrace from authorities and the public, a report or document from an office like the police or health facility is important. This document can later be used in the death registration process
	Interpersonal (friends/family and social networks	Seeking aid	Families and children of the deceased can seek aid in form of bursaries and scholoarships for study. Having a death certificate gives them advantage in such applications for aid
	Identity	Identity of the deceased	know the right identity of the deceased
1	People / individual	Having the necessary documents	The LC letter and minutes from the family meeting
		Knowledge about death certification	Some people know that it is a requirement to get death certification after the death of an individual
		Perception that is only for those that had properrties	Right administrator who will develop such property. Now, for us who don't have property, it is done after burial, there is no registration.
		Knowledge about certification	Having Knowlegable about the process
		Have the telephone contacts of the officers that you need	Have contacts of the officers that you need in the registration process. They dont sit in their offices all the time so you may need to call them first before you go their
		Awareness of relevant stakeholders about the death reporting process	Stakeholders such as health and community health workers and local leaders are informed that death registration should be done

		Histroy of death registration attempts	Members who have ever attempted to register death were informed about the registration process
		Presence of close (the nuclear) family members	Close family members include children and spouses of the deceased. Especially for an only child, processing a death certificate will not be hard since he/she is not fighting with anyone for the remaining assets of the deceased
		Presence of a will	The will makes it easy to process the death certificate especially without wrangles in the family. The deceased's family will always follow the wishes of their late loved one
2	Human resources	Availability of the officers in charge of reporting and registration.	The LCs, subocunty chief and NIRA officers are stationed at the district headquaters
		Competent training of the health workers	The health workers are adequately trained on all the processes, their roles and forms required for reporting and notifying death.
		Some knowledge on death reporting	Health workers must report deaths in facilities via HMIS reports, noting the cause of death, treatment, and patient details. The registers record patient information, including deaths, but primarily list the cause of death.
		Presence of Health local leaders in communities and in field	The presence of HF staff in community eases the report of community deaths since they do not have to incur transport costs int to notify. Also, loved ones barely remember to notify death immediately after their beloved have passed on. they are mourning, so informing authorities is not a priority. The presence of HF staff in field/community makes it easy for boths to communicate.
		Presence of readily available lcal leaders	Local leaders such as the VHTs and LC1 are always available to give a hand in community when called upon by the police and health facility
		Presence of leaders in the area	The LC and subcounty chief reside in the same area which makes the process easy
		Involvement of local leaders in death notifcation	When VHTs are involved, they always inform the Chairperson. The 2 are community initiators in death notification to higher levels such as the subcounty. Notifying them eases the notification of higher levels to initiate and propel the death registration process.

		Presence of marine police on ground	These are located on the habitable islands of the district and always confirm deaths before the district authorities get involved
3	Information	Report/ record keeping	its important to always keep records at national level, that someone pased away for planning purposes
		Disease surviellence	Helps to know which diseases are people dying of
		Outbreak alerts	It is upon us as chairpersons because we continue to inform our people that there is death in a certain area or house.
		Good reporting systems at the facility	The health facility policy of recording and registering people who have died at the health facility makes it easy
		Availability of notification forms/registers	Having the registers allows for keeping of written evidence of someone's death
		Contact information shared by stakeholders	Health facility staff always share their contact information for easy access and comunication with the community
		Presence of different means of communication	Communities and stakeholders are informed about death and related information through traditional media such as radio, and social media platforms such as whatsapp
4	Financing	Availability of funded needed for the process	That includes money needed to pay for the certificate and money for transport to the different offices
		Affordability of the death notification process	No payment is required for the death notification form or the postmortem examination procedure
		Presence of emergency funds at the health facility	These funds are used to cater for the deceased since there is no clear plan for their care especially in lower Health facilities.
		Cheap costs of death registration	Some have witnessed the registration process to be cheap and free unlike in the past
5	Service delivery	Government planning purposes	Death registrations helps the governement to plan for people who are living
		Nira proximity	The NIRA offices are now near to the community

		Issuing of a death report	When healthcare workers make a cause of death report, its easier to process the death registration because the reports are legit reports.
		Geographical location of instituitions	The close proximity of the police post to the health facility eases the deceased's family to get the required documentations from both offices in a short time
		Long working hours of relevant institutions	The police and heatlth facility are open 24/7 which makes it accessible by the deceased's family at any appropriate time of the day
		Efficient registration process at NIRA	The process has now become faster
		Easy access of community to health facility staff	Community can inform whichever HF staff is of convinience to them for death notification
		Familiarity	The LC is familiar with the deceased's family
6	Supplies and technology	Availability of equipment and materials to use	NIRA has provided laptops to Health Centre IV to enable them enter mortality data. The health workers also have books needed for death notification and registration
		Presence of official death recording and notification templates	These are requested by NIRA to process the death certificate. The forms are present both at HF and the police but these might be unsyced which bring data compilation challenges. Perhaps the 2 parties dont share their data with each other.
7	Governance and leadership / policy	Government requirement	Government requirement to know how many people are born and how many die.
		Presence of proper structures	When someone dies at the facility, the health workers know where to report
		Follow up and monitoring	NIRA does routine check-ups at the facilities to monitor whether the deaths are being reported. Ministry of Health also organises weekly meetings to track women and newborns who die
		Strong collaboration amongst the key stakeholders	There is good collaboration between the key stake holders involved in the process of death reporting, reporting and registration, police, active community health workers, NIRA sub county officers and facility health workers.

		Knowledgeable local leaders	Leaders who are well versed with the process of death notification are in position to advise and guide people on death notification
		Presence of death notification guidelines	There are highlights of what to do at the health facility regarding notification and reporting of death and this has helped health workers to notify deaths along their hierarchies
	Government policy		Registration helps government know the cause of deaths in the communities
8	Community / Culture	Presence of community health workers/leaders	Community health workers brief health facility workers about community deaths
		Perceived benefits	The community members believe that death registration they can get some gains from registering deaths. They also believe that death registration is important because it helps the government plan accordingly.
		Awareness raising at community level	When messages concerning the process of death registration are passed over community radios, more people are able to know and embrace the process
9	Interpersonal (Family/friends/ social networks		
		Support from the local leaders	The local leaders gave us a letter to start the process
		Support from relatives	I have a sister who works at the district and also the chairperson is my brother, they are supporting me throught the entire process
		Support from the family	Family meetings are held and minutes taken down, selected a successor to follow up on the process.
		Need for adminitrative documents	Registrations mainly occur for those with property to avoid family disputes. Administrative documents are needed to determine property ownership and heirs.
		60% of Kalangala's population aren't natives.	60% of Kalangala's population aren't natives.
		Training facility staff	It was noted that training of staff on the process of death notification was a way towards improving their skills and knowledge around registration of deaths

		Good collaboration between stakeholders	NIRA, Health facilities and the police woork hand in hand to ensure death notification and registration.
		Teamwork between health facility staff	In the absence of the health facility incharge, his/her subordinates conduction the death notification form. The health facility always gives feedback to the VHTs about the death of their community members
		Effective communication with supervisors at hier level	
		Good relationship among family members	This eases the death registration process because they are in one accord and so are the documents they present to the different offices.
		Good relationship of the deceased's family with local leaders	If the relationship between these parties is good, the local leader can easily give a hand in death notification to the relavant stakeholders and advises on how to register death
10	Others		
		Registration books	Community health workers improvied registration books to keep track of every person who dies each year
		Understanding of death registration	Death registration means surveillance of any dead in community for proper allocation of health services. Importance of death registration is; for organised service delivery
1	People /individual	Limited information	People dont know about death notification and they dont know where to start from,
		Lack of Knowledge	with the little knowledge the LCs have from the past 10years training. even if we start emphasising it to the community memebers, they will think ist for our own benefit. thats why its upon only those who see the benefit of it that they approach the LCs.
		Ignorance	Some people are totally not aware about death certification
		Unaware of the registration services	People are unaware of the registration services at NIRA
		ignornance	people arent aware of the importane of death registration

		No benefit to registering a dead	People do not find any benefit or purpose why they should register and have a death certificate of the decessed.
		Managing the deceased’s property can get personal	Managing the deceased’s property poses significant challenges due to corruption, so people resort to giving up
		Perceived risks	People tend to fear high costs that might be incurred during the whole death registration and notification process. Additionally the community members fear they might be asked a number of questions about the death.
		Emotional distress	The grief due to the loss of a loved takes a toll on the relatives thus preventing them from notifying and reporting the deaths
		Fear of reporting a violent death at the police	People fear police delays and questioning, leading some to bury deceased villagers without reporting to avoid prolonged investigations.
		Fear of legal implication	Participants noted that if they reported a death, they would be asked a lot of questions about the cause of the death, or even be imprisoned
		No perceived importance of death certificate	A concern was noted that if for example, it is a child who has died, then there is no use of processing a death certificate for them
		Knowledge limitations	The people don’t know and have never heard about the death reporting, notification, and registration process
		Lack of interest	People are lazy, don’t care and deliberately refuse to register deaths if they have nothing to gain.
		Lack of Knowledge	People dont know abouth the death notificstion process.
		Poverty	Some families are too poor to transport the body of their loved one to the Health Centre for postmortem
		Time consuming and bureaucracy	The process takes long and people feel it is not worth their time given that someone died. Additionally, the authotrities ask for many documents which makes families give them in the long run
		Poor Knowledge	Individuals have poor knowledge about the death registration process. Others do not attach value to it because of their poor knowledge.

			Poor knowledge about the importance of death registration and certification
			No knowledge of the existence of a death registration authority and the process. Some people were hearing of it for the first time
		Negative attitudes towards registration	People who register the dead may steal their property
		ignorane	people are not aware of the importance of registration and others arent aware that NIRA registers the dead
		No financial benefit	Relatives arent willing to register the dead if there is no financial benefit attached
		Privacy	Some women don't want to expose the right paternity of their children especially if they had multiple partners and aren't sure who the right father is.
2	Human resources	Unavailability of officers responsible for registration	The officers are not always available at thier offices when needed
		Negative atittude	The officers have an i dont care atittude
		Lack of awareness of cultural leaders	Authorities are not sensitized and don't know what to do.
		Failure by the health workers to report death on time	Some health workers take long to report the deaths that occur. Deaths should be reported weakly
		Lack of training among LCs	That system has not been taught to us and we don't know
		Bureaucratic, lengthy, and inefficient death registration.	The death registration process is lengthy and bureaucratic, involving hospitals, communities, police, and NIRA. Delays occur due to healthcare worker shifts, busy NIRA officers, and the need for multiple family confirmations.
		Gaps in staffing	There is absence and shortage of workers in charge of death notification and reporting at both facility level and village level.
		Poor work ethic	The community and facility health workers and sub county officers are neglecting their duties and overall displaying an "I don't care attitude". The community health workers are focused monetary gain instead of their work

		Partial training of Healthcare workers by NIRA	NIRA's training was limited, with only a few focal persons trained, mainly in maternity. Most general health workers lack comprehensive awareness about death reporting. A comprehensive training for all health facilities is needed.
		Limited personnel	There were few trained personnel equipped with the technical capacity to handle the process of death notification
		Training gaps	There is lack of training among health care workers, local leaders, community health care workers regarding the death reporting, notification, and registration process.
		Post-mortem cases from the communities are assocaited with negative reactions from the community	Only medical officers or doctors can conduct post-mortems, with senior clinical officers in some cases. Community and police often misunderstand this, causing negative reactions when workers can't help. Community deaths not related to public health are rarely reported in the system.
3	Information	Out dated death registers	Old death registers that were last supplied in 2007
		False information	Informaion given to LC1 is forged
		NIRA's roles are not felt at grassroots	It is responsible for updating records
		Data inconsistencies	There are discrepancies between the death records at NIRA and records at the health facilities. Findings also show that people receive death notifications from sub counties in which they don’t reside thus complicating capture of mortality data.
		Documentation requirements	Alot of paperwork is needed for the death registration process
		Poor network/internet connectivity	Network challenges posed a barrier to updating of the dashboard in instances where registration is to be done
		Identity	Lack of enough information about the deceased
4	Financing	Certification fee	Some people do not see any benefit in paying for the certificate for a person who is never coming back to life

		Lack of finacial facilitation for VHTs	VHTs lack transport facilitation to support death registration
		The process is too expensive	The process of moving from one level to another costs alot of money in regards to persons your to meet, transportation costs and the certification fee, forexample the prganisations like the Uganda human Rights commission asks for money to process the death certificates
		Poor motivation	Their is no motivation for the VHTs to carry on with the process
		financial constraints	people dont have the money to follow up on the process
		Process is paid for	People do not have the money they ask for to register
		Being charged to get a notification document	Participants noted that local council authorities ask for money for them to be able to push the process of death notifiction and registration
		Increase salary and timely payments	Increase our salary to improve our work quality and reach more areas.
		Limited access to financial services	Without nearby banks on the island, the community members face a challenge of paying for the certification fee.People also lack the money to pay for the death certification fee and the mortuary fee thus smuggling the dead bodies.
		High costs	High costs incurred in the death regsitration and certification process. Death notifcation at the Health Facilities is also expensive since money is required to transport the deceased for postmortem. Given that there is no clear plan for the Health Facility to transport the deceased for and after postmortem, the burden falls on the deceased's famility, which the family can ignore to do to prevent incurrance of costs.
		Limited facilitation	Both the VHTs and Health facilities are underfunded to conduct death related activities/services.
		Potential bribes involved in the death registration process	Communities percieve that the process becomes long due to the money that has to be informally paid to the difference offices in the chain

		Long distances	people do not have money to go through with the process of registration. people need alot of transport from other islands to get to Kalangala town council
		financial constraints	Alot of money is needed to be able to register. that includes money for transport and bribing officers
5	Service delivery	Bribery unto receiving services	Some health facilities require you to pay some money if you want to be assisted with support letters on time to proceed with the registration/certification
		Corruption	Managing the deceased's property is challenging due to bribery, making many people give up.
		Corruption for services	People lack the money to pay for the death certification fee and the mortuary fee thus smuggling the dead bodies.
		Absence of officials	They are not residents of the island
		High corruption rate	Corruption is a major issue, forcing people to pay bribes at every step when registering a death. This creates financial hardship and can prevent completing the process. The situation worsens if the deceased owned property, making it seem like a trade to register the death.
		Long distance to access the services	Participants noted that the services were from where they were and thus would have to travel long distances just to go and follow-up with the notification and certification process, whch was not feasible
		Health care access hindrances	The island lacks health care facilities to carry out the notification process and the poor road conditions especially during the rainy seasons, make it difficult to access the health care facilities that are available.
		Poor communication network	Phone communication is a challenge at some islands of the district. This results into missing information for both the communities and stakeholders hence hinders the death notification and registration process. This demotivates people from incurring an extra burden to conduct postmortem for their deceased, hence no reports will be recorded at the health facility. Such cases will just be buried without even notifying the police.

		Bias of health workforce	Health local leaders such as VHTs, Health assistants and health faciltiy staff are more inclined to reporting death only to their hierarchical authorities such as the HSD manager, the surveillance focal person and this is mostly for disease related deaths. They are more focussing on disease related deaths with an aim of preventing outbreaksThey will barely report to NIRA
		Community deaths are barely noticed by stakeholders	Health local leaders suspects some deaths to go unnotified
		Corruption	Officers ask for money and they work easily on families that have alot of property because they will gain
6	Infrastructure, Supplies and technology	Unavailability of documents	The local leaders dont have the materials needed for death notification and reporting
		Poor accessibility	The fact that people must rely on water transport all the time, it limits access to some hard-reach areas. Therefore, such people can not benefit from some of the government services all the time
		Shortage of supplies	They lack the technological devises to use in registering
		Limited equipment for death registration	Participants do not have enough laptops to store the data electronically
		Lack of formal tools for reporting deaths outside health facilities	We lack formal tools for reporting deaths outside facilities, missing system capture; informal methods and police post-mortems exist.
		Lack of death notifications at the Health facilities	NIRA, responsible for vital statistics, planned health worker training years ago, but only a few were trained. Facilities handle birth and death notifications, but lack death notification forms.
		Power shortages	Different institutions in charge of death notification and registration may face power outages which delays service delivery. This demotivates people from continuously physically visiting them for deth reported reports to start the registration process.

		Unaligned documents	The death notification templates at the police and the health facility are different from eachother yet both important. Being at different locations becmes more work for the deceased's family to follow up, especially if the two institutions are located farm from each other.
		Unsynced system	The VHTs are not directly included in the loop of death notification workflow, yet are always consulted by community.
		Lak of forms	There are no specific forms where the dead are registered
7	Governance and leadership / policy	Poor leadership	The leaders at the health facility are not informing the community about death notifications and reporting
		Lack of collaboration between the relevant stakeholders	There is no collaboration between responsible authorities such as police, NIRA, health workers
		Its bureaucratic and a long process to pertake	it's a long process, which requires moving up and down
		Lack of facilitation	Authorities that are responsible to support death notification and registration are not facilitated which hinders engagement such notification activities
		Failure to implement	Leaders do not implement government programs
		Limited support supervision/ monitoring by the relevant authorities and leaders.	Leaders don't follow up to see whether the health workers are carrying out the death notification and reporting process as per the training
8	Community / Culture	Lack of knowledge	Communities lack knowledge on death registration, and the responsible authorities
		Misconceptions	Community members think when they are called upon to register deaths, it is the local leaders who gain, and therefore decide to conceal information.
		Mobile population	Most people in the island are not naives they just come for work
		Cultural and religious influence	It was noted that death notification and registration may not necessarily align with some cultural traditions

		Lack of gains	People prefer if they physically gain from the registration of the dead
		Community intruders whoo have no identity in the community	Some people come and die in communities when no one knows about their family background in order to follow-up with them for death registration.
		Time wasting	Some people look at notifying deaths as time wasting
		Failure for the community to follow up	Some community members think that it is the role of the health workers to register deaths. They dont bother to proceed unless asked by the health workes
		Poor perceptions towards registering	Community views registration of the dead as uneccessary, if they are dead, they are dead, they cannot add onto the community
		Misperceptions of death registration	Community members believe that death reporting, notification and registration is unimportant since the deceased are no longer useful. They also lack awareness about the whole process thus take it as unserious.
		Registration seen as property theft or implying guilt.	Some perceive registration as a threat, thinking it's about property theft or implying guilt, contradicting traditional beliefs like "the dead cannot be counted." They see death registration as unnecessary.
		Religion	The Islamic culture has a tradition of burying the dead instantly thus in most cases the burial ceremonies are held without first obtaining the death notification
		Cultural norm	The Buganda culture perceives death registration as counting the dead which is a aganist the cultural norms. though its only knoen by the clan leaders and cultural leaders.
		Ignorance about Death registrations	People lack awareness about registration procedures, especially for deaths. There's limited understanding and no follow-up,increasing ignorance.
		Insecurity	Communities are afraid of initiating the registration process due to fear of being associated to the illegal activities of their deceased loved ones especially if they were criminals.
		Immediate burial	Communities know and believe that right after death of a loved one, the next is a burial ceremony. This is very common in the muslim faith

		Cultural hindrances about the dead	Communities believe that it is not good to talk about the dead, therefore always discourage the close family members to proceed with certification. Communities also believe that the organs of their loved ones will be harvested from the morgue during post mortem
		Hidden identities	Communities know that islands are hiding places and safe spaces for criminals, and know that the known name of an individual might not be their real name. Upon death, it is difficult to claim bodies of deceased who had hidden identities in their waking lives. As a result, their bodies are disposed of/buried in communal/public burial grounds.
		Negative perceptions	
9	Interpersonal (friends/family and social networks	Disagreements	
		Family issues	Families failure to decide who takes responsibility
		Family disagreements	Wrangles within the family are a source of hindrance in the death registration and certification process. This is due to varying documents that could be presented to the officals about the same deceased relative.
		Non-Ugandan families	This is becuase claiming ownership of the body and its postmortem report means that you have to transport the body for burial in the ancestral land out of the country. This comes with financial implications which the families are not ready to incur.
10	Others	Lengthy process	The process of acquiring a death certificate is too long, and that demotivates people from chasing for it.
		Not prioritized	Some people do not take acquisition of a death certificate as being important so they do not give it priority
		Cause of death	Some people violently die without any information talking about them and not easy to identify, which makes the follow-up on their death certificates impossible.
		Intruders	Some people go into the islands without any information that talks about them. So, when they die, no one is able to follow-up on their death certificates

		Long process	The death registration process takes long
		Lengthy process	The process takes alot of time to accomplish
	**People/ Individual**	Intensive community sensitization	The community needs to be sensitized about the importance of death registration
		Strengthen death registration	The facillity health workers need to sensitise the community about the importance of death registration and reporting
		Support orphans	There is need for relatives to support orphans to get the certificate
		The need for sensitisation about death registrations	The people need to be sensitisedon the importance of death registrations
		Community sensitisation	They should be sensistised about the existence of death certification, its importnace and the steps to go through the process
		Sensitisation of communities with the help of parish chiefs	People have more confidence in the subcounty parish chiefs when they play their roles on sensitising communities on what should be done
	**Human Resources**	Training human resource	government should train the VHTs and cultural leaders on the technical aspects of managing the process of death registration in the communities professionally
		Training of the responsible authorities	Health workers need to be trained about death notification, reporting and certification
		Intersectoral collaboration	The relevant bodies and autorities should work hand in hand in promoting awareness of death notification, reporting, and registering
		Motivation, training, and providing protective gear	Police officers also need to be motivated, trained and availed protective gears
		Health workers role in the cascade of death registration s should be enhanced.	Train health workers at facilities to stress the importance of death registration, aiding census accuracy and reducing costs.

		Sensitisation and awareness creation	NIRA's mandate is to train the local authorities such as bthe chairpersons, VHTs, youth leaders to train communities on the importance of death registrations and the process of acquiring them
		Sensitization by CDOs, chiefs, and community groups.	Sensitization can be done by CDOs, parish and sub-county chiefs, and committees of women and youth groups through village visits, flyers, and massive mobilization, explaining the benefits and process of death registrations.
		Use of case studies of those that benefited from death registrations	we should use case studies to highlight the benefits of registration versus not having a certificate. Implement a quick process for registering and issuing death certificates before burial. This serves as proof of death and prevents cases like a community mistakenly burying the wrong person, as I once witnessed.
		Sensitisation and awareness creation	Sensitize communities about death registrations through training, burial ceremonies, and places of worship, emphasizing its importance and consequences.
		Increase on the human resources	Employ an officer responsible for death registration.
		Provide Trainings	Provide refresher trainings to health workers and VHTs about the importance of death registration
		Enhance NIRA services for accurate registrations.	If the government enhances NIRA services, everything will improve. NIRA must register births and deaths, requiring government support. Continuous training, especially in villages, is essential. NIRA should proactively inform and engage communities.
		Train VHTs	train VHTs on the technical capacity of handling registration in communities
		Facilitate and support VHTs	Provide VHTs with an allowance to ease their work
		Train VHTs	To strengthen their capacity
		Priotise death notification	Govement workers should give priority to notification
		Staff turn over	Inactive community health workers should be replaced
		Engaging other village local leaders	The parish chiefs, the LC1s, LC2s, the VHTs and the health assistants all need to be brought on board to improve death reporting, notification, and registration.

		Sensitization of the key stakeholders	Sensitization of facility health workers, community health workers, local leaders about the process, how to enter mortality data into the system, the forms to be filled and the importances of death reporting, notification, and registration. CMEs and also audits should be continuously carried out to improve death reporting.
		Train VHTs	There is need to train the community, LCs, and VHTs
		Specific focal person should be assigned the death registration	Designated people need to be put in charge of the death reporting, notification and registration at the health facilities and the NIRA offices.
		Continuous mentorship	Regularly conduct mentorship workshops for providers at the facility to build their capacity.
		Make system accessible to all personnel	All data personnel should be able to log into the system to make death notification and registration entries
		Sensitisation of human resource about the details of death registration and certification	These include VHTs, Health workers, CDOs. Their roles should be well described. Health Workers can be sensitised through Community Professional development sessions or CMEs
		Reduce health worker workload	This can be done by increasing staffing at the facility
	**Information**	Utilizing the available infrastructure to promote awareness of death notification, reporting, and registration	The responsible authorities should conduct the community sensitization from the health facilities because they are accessible to everyone
		Records	Provide books or tabs/phones where to keep records of people who have died
		Digitalise the system	Provide tablets or phones where to store information
		Provide manual	Provide a guide to the Local leaders as a guide on the notification process

		Sensitization campaigns through different social media outlets	The government should create awareness about death registration through TVs, Radios etc.
		Research	Academia should do more research about death reporting, notification, registration and certification
		Senstisation of the masses and the leaders	Senstise the leaders and the people on how to notify because the leaders are always replaced
		Improved communication	NIRA should improve communication to the lower authorities and communities about the process and importation of death registration and certifiation
		Information sharing	Health facilities should provide death information to NIRA
		Community death recording	VHTs should also have death records
	**Financing**	Financial facilitation of the responsible authorities	The responsible authorities need to be facilitated in terms of transport refund to access all areas and support death reporting.
		Issueing of Death certificates at the points of death	The two points of death, at healthcare facility an at community level. There should be forms of death certificates
		Make people aware of the funds needed for registration	People should be made aware of how much they need to complete the process of registration
		Allowances	The community and facility health workers should be given allowances to boost their morale to aid in the process death reporting, notification, and registration.
		Financial support to VHTs	
		Result oriented income	Programmes should be introduced whereby, the more death registartions the health facility carries out, the more money it gets,
		Allocate funds to the process	Budgetary allocations should be set aside to aid in the process of death registration
		Facilitation	Local leaders such as VHTs should be facilitated to guide community members through the process of death certification

	**Service Delivery**	Work together	strengthen the police's reporting system
		Free the process	Need for all the people involved in death registration to work together
		Collaboration of the different stakeholders to achieve this purpose of death registrations	Let the process be free from any cost charges
		Bring services to the grassroot level	Registration should be done at grassroot level the VHT and LC
		Improving accessibility	Death registration should be scaled down to parish and village level.
		Decentralise the registration process	Bring services to the subcounty so that they are easily accessible by everyone
		Follow up	NIRA should follow up
			District collaborations with the different stakeholders
		offer assitance	Offer transport to those who cant afford to transport bodies to and from the community
	**Infrastructure, Supplies & Technology**	Availing of death regiters at community level	The certificates should be brought at the health facility and subcounty level
		Registers	providing death register forms to all LCs for verifications
		Issuing death notification documents at village level	Health facilities should give VHTs registers to register deaths in communities
		Providing health facilities	Community members should report to the local and cultural leaders who should give a written and signed documents to the notifiers
		Provision of tools and resources	Kabaka to provide more health facilities to the community
			Provide us with tools and resources to empower and facilitate our work for the government.

		Digitization of the death reporting, notification, and registration process	The death registration should be digitized. Mortality data being reported by the health facilities and sub counties should also be digitized
		Print media	There is need to provide death notification forms to the LCs to ease notification. Manuals and guides should be printed and distributed to the community with the whole process of death reporting, notification, and registration.
		Usability of the dashboard	There is need to make the dashboard more user-friendly
		Construct a mortuary at lower Health facilities	This will encourage communities to take bodies for record
	**Governance/ leadership**		
		Priotise death notification	Government workers involved in politics must give priority to death notification
		Making death certification compulsory	Death registration should be extended to village level to minimize expenditures on transport, and people's time
		Shortening the registration process	A law should be passed to make it a must for every death to be registered
		Facilitation of the responsible authorities	Certification of the deaths should be made shorter to ease acquisition
		Make it compulsory	Responsible authorities should be financially supported to run the relevant tasks in regards to death notification and registration
		Government Facilitate	Government should front the process like it does on birth certificate
		Enforce the law	send law enforcers at grassroot level
		Transparency	The collaboration betweens takeholders should be transparent
		Decentralize the registration process	Making it mandatory for everyone to register death
		Senstise at burial place	Leaders should senstize the bereaved family
		Sensitise people	Let the registration process start from the community
		Multisectoral collaboration	Government should sensitize people about the benefits of registering deaths.

		Instant documantation	Sesitization of people and pushing for death registration and certification should not be one-sided, only inolving a few authorities/bodies, but it should involve all the relevant authorities for maximum adoption
		Enforce laws	Get the death report document signed by the local leaders immediately a person dies
		Bring services close to people	Ensure the law on death notification is enforced from the lowest administrative unit
		Enacting death registration law	Laws should be put in place clearly stipulating all the death registration procedures and mandating the death reporting, notification, and registration process
		Prioritization of death registration	The government needs to prioritize death registration process
		Collaboration among the key stakeholders	The local leaders, politicians, the community health workers, and facility workers need to work hand in hand in order to improve the death reporting process.
		Enforce the law	Government shoud come up with a law making it mandatory to register people who have died
		Continuous supervision/ monitoring	NIRA and the DHOs need to carry out constant follow up at sub counties, villages and health facilities to ensure that death registration is being carried out.
		Decentralisation	This includes decentralising power and authority to lower subcounties and to lower local leaders such as the VHTs regarding death certification. It will reduce the transportation costs incurred
		Collaboration with different stakeholders	Every stakeholder has a role to play to ensure death registration and certification. Working with religious leaderships is an example
		Guidelines about death registration fees	The authorities should establish and publisize a known fees incurred at the different levels during death registration
		Identification of the unknown bodies	Notification of peeople who died with hidden identities should be done
		Immediate initiation of the death registration process	Guidelines should be established to ensure that the deceased are registered as soon as possible
		Enforcement of sensitisation	Sensitisations for death registration should have a budget and should be enforced

		Guidelines about bribes and corruption	Enforcements should be done to reduce corruption and bribes during death registration
		Guidelines for lower Health facilities	These should be reviewed to provide supplies to HCIIIs that are required in care of the dead. This will motivate people to bring their deceased to the facility for record
	**Community/ Culture**		Bring registration services into the community
		Community sensitisation by LCs	NIRA should sensitize communities about the processes of registration.
		Sensitize and bring services closer	Because every death at the village, LCs have to be notified, so LCs are good stakeholders to preach the death registration, since they are almost know everyone in their communities.
		NIRA should proactively register births, deaths.	Sensitize communities and bring registration services closer to them
		Mass death registration	Mass death registration should be carried out across the whole country.
			NIRA only registers what is brought to them. They should proactively visit villages for registrations, not wait for school requirements. Parents often neglect registering children until required. Government funding can help NIRA fulfill its mandate to document births and deaths accurately. Currently, NIRA's reliance on schools for information has led to issues with incorrect data, causing problems for parents.
	**Interpersonal**	Stakeholder engagement	When stakeholders are involved in the process, it helps make it much easier for the community to accept, when they go talk to them
	**Others**	Media engagement to help improve awareness	Participants recommended that engaging the media teams, either through radio or TV, to disseminate information about death notification would be helpful

