# Supplementary material for: Experiences and Perceptions About Death Reporting and Notification Among Rural Communities on the Islands of Lake Victoria, Uganda: Qualitative Study
Source: JMIR Public Health Surveill. 2025 Aug 13;11:e77135. doi: 10.2196/77135 (PMC12347208; doi:10.2196/77135)
Supplement: Checklist 1 [file publichealth-v11-e77135-s002.pdf]

Manuscript: Experiences and Perceptions About Death Reporting and Notification Among Rural Communities on the Islands of Lake Victoria, Uganda: Qualitative Study

Consolidated criteria for reporting qualitative studies (COREQ): 32-item checklist

Developed from:
Tong A, Sainsbury P, Craig J. Consolidated criteria for reporting qualitative research (COREQ): a 32-item checklist for interviews and focus groups. *International Journal for Quality in Health Care*. 2007. Volume 19, Number 6: pp. 349 – 357

No. Item	Guide questions/description	Reported on Page #
**Domain 1: Research team and reflexivity**		
Personal Characteristics		
1. Interviewer/facilitator	Which author/s conducted the inter view or focus group?	Page 9, under data collection
2. Credentials	What were the researcher's credentials? E.g. PhD, MD	Page 9, under data collection
3. Occupation	What was their occupation at the time of the study?	Page 9, under data collection
4. Gender	Was the researcher male or female?	Page 9, under data collection
5. Experience and training	What experience or training did the researcher have?	Page 9, under data collection
Relationship with participants		
6. Relationship established	Was a relationship established prior to study commencement?	Pages 10, under ethical considerations
7. Participant knowledge of the interviewer	What did the participants know about the researcher? e.g. personal goals, reasons for doing the research	Pages 10, under ethical considerations
8. Interviewer characteristics	What characteristics were reported about the inter viewer/facilitator? e.g. Bias, assumptions, reasons and interests in the research topic	Page 9, under data collection and Page 10 under ethical considerations

**Domain 2: study design**		
Theoretical framework		
9. Methodological orientation and Theory	What methodological orientation was stated to underpin the study? e.g. grounded theory, discourse analysis, ethnography, phenomenology, content analysis	Page 7 under study design
Participant selection		
10. Sampling	How were participants selected? e.g. purposive, convenience, consecutive, snowball	Page 7, under sampling and sample size
11. Method of approach	How were participants approached? e.g. face-to-face, telephone, mail, email	Page 9, under data collection
12. Sample size	How many participants were in the study?	Page 7 under study population and sample size and sampling
13. Non-participation	How many people refused to participate or dropped out? Reasons?	Not applicable
Setting		
14. Setting of data collection	Where was the data collected? e.g. home, clinic, workplace	Page 9, under data collection
15. Presence of non-participants	Was anyone else present besides the participants and researchers?	Not Applicable
16. Description of sample	What are the important characteristics of the sample? e.g. demographic data, date	Page 9 under study population and sampling
Data collection		
17. Interview guide	Were questions, prompts, guides provided by the authors? Was it pilot tested?	Page 8, under data collection
18. Repeat interviews	Were repeat inter views carried out? If yes, how many?	Not applicable
19. Audio/visual recording	Did the research use audio or visual recording to collect the data?	Page 9, under data managament and analysis
20. Field notes	Were field notes made during and/or after the inter view or focus group?	Page 9, under data collection
21. Duration	What was the duration of the inter views or focus group?	Page 9, under data collection
22. Data saturation	Was data saturation discussed?	Page 9, under qualitative data collection
23. Transcripts returned	Were transcripts returned to participants for comment and/or correction?	Transcripts were not returned to participants for comment or correction due to

		logistical constraints and time limitations. However, efforts were made to ensure accuracy through verbatim transcription and note-taking during interviews.
**Domain 3: analysis and findings**		
Data analysis		
24. Number of data coders	How many data coders coded the data?	Pages 9, under data management and analysis
25. Description of the coding tree	Did authors provide a description of the coding tree?	Not applicable
26. Derivation of themes	Were themes identified in advance or derived from the data?	Pages 9, under data management and analysis
27. Software	What software, if applicable, was used to manage the data?	Not Applicable
28. Participant checking	Did participants provide feedback on the findings?	Findings were not formally validated with participants after analysis; however, triangulation across multiple data sources and use of a structured coding framework supported the credibility of results.
Reporting		
29. Quotations presented	Were participant quotations presented to illustrate the themes/findings? Was each quotation identified? e.g. participant number	Results Pages 10 to 15
30. Data and findings consistent	Was there consistency between the data presented and the findings?	Results Pages 10 to 15
31. Clarity of major themes	Were major themes clearly presented in the findings?	Results Pages 8 to 15
32. Clarity of minor themes	Is there a description of diverse cases or discussion of minor themes?	Results Pages 10 to 15
